# Fact or fiction - identifying the elusive multiple myeloma stem cell

**DOI:** 10.1186/1756-8722-6-91

**Published:** 2013-12-07

**Authors:** Joshua Kellner, Bei Liu, Yubin Kang, Zihai Li

**Affiliations:** 1 Hollings Cancer Center, 29425 Charleston, SC, USA; 2 Department of Microbiology and Immunology, Medical University of South Carolina, 86 Jonathan Lucas Street, 29425 Charleston, SC, USA; 3 Division of Hematology and Oncology, Department of Medicine, Medical University of South Carolina, 86 Jonathan Lucas Street, 29425 Charleston, SC, USA

## Abstract

Multiple Myeloma (MM) is a debilitating disease of proliferating and malignant plasma cells that is currently incurable. The ability of monoclonal recurrence of disease suggests it might arise from a stem cell-like population capable of self-renewal. The difficulty to isolate the cancer stem-like cell in MM has introduced confusion toward this hypothesis. However, recent evidence has suggested that MM originates from the B cell lineage with memory-B cell like features, allowing for self-renewal of the progenitor-like status and differentiation to a monoclonal plasma cell population. Furthermore, this tumor-initiating cell uses signaling pathways and microenvironment similar to the hematopoietic stem cell, though hijacking these mechanisms to create and favor a more tumorigenic environment. The bone marrow niche allows for pertinent evasion, either through avoiding immunosurveillance or through direct interaction with the stroma, inducing quiescence and thus drug resistance. Understanding the interaction of the MM stem cell to the microenvironment and the mechanisms utilized by various stem cell-like populations to allow persistence and therapy-resistance can enable for better targeting of this cell population and potential eradication of the disease.

## Background

Stem cells are classified as cells that are pluripotent and can propagate the cells of a specific lineage while also maintaining self-renewal. Recent evidence has suggested that cancer has exploited this unique machinery and contains a stem-like population that maintains and propagates disease. The current paradigm regarding the cancer stem cell (CSC) is that the tumor either arises from a normal stem cell or inherently contains a “tumor” stem cell that drives tumor formation. However, it is debatable if these paradigms can apply to all cancers or if they are unique to several specific cancer types. Two initial studies laid claim to the hypothesis of CSCs. Bergsagel *et al*. characterized a low frequency population with tumorigenicity in a plasma cell tumor model of inflammation which led to studies where murine myeloma cells, isolated from ascites, could form *in*-*vitro* colonies at a ratio of only 1 in 10,000 to 1 in 100 cells [1,2]. Various solid tumors, such as lung and ovarian cancer, also exhibited a high degree of tumor-initiating heterogeneity with only a small subset of the tumor population exhibiting clonogenic potential [3]. The development of an *in vitro* assay to study human myeloma clonogenic cells furthered the work delving into the hypothesis of a progenitor cancer cell [4]. However, it was the work from Dick and colleagues who identified a CSC from a specific subset of acute myelogenous leukemia (AML) cells that had demonstrated the clonogenic activity of a particular isolated population with confidence [5]. This minute population, demonstrating a varied frequency of about 0.2% in some patients, was capable of transfer disease into immunodeficient NOD/SCID mice. These studies suggest the presence of a CSC but it is difficult to determine whether they are generated from a mutational hit on normal stem cells or from a specific primitive tumor stem cell. The ability to isolate primitive hematopoietic stem cells (HSC) and our understanding of the stem-like mechanisms of HSCs has enabled better understanding of CSCs in leukemias but has proven to be more difficult in myeloma.

## Pathology of disease

MM is an incurable blood malignancy characterized by extensive proliferation of plasma cells (PC) and displaying an incidence of about 20,000 annually in the United States [6]. The tumorigenic PCs secrete monoclonal immunoglobulin and induce a wide range of pathology including lytic bone disease, hypercalcemia, immunodeficiency, anemia and kidney and bone marrow (BM) dysfunction [7]. Nearly all MM patients derive from asymptomatic monoclonal gammopathy of undetermined significance (MGUS). Patients could present as smoldering MM phase that then progresses to advanced symptomatic phases of MM, which include an active, relapsing and refractory periods [8]. Various treatments for MM have been developed including corticosteroids, DNA alkylating agents, immune-modifying drugs, proteasome inhibitors and hematopoietic stem cell transplantation (SCT). Over the last decade, the overall survival of MM patients has improved from a median of 3–4 years to currently at 5–7 years, largely due to the use of several highly active chemoagents and the incorporation of autologous HSC transplantation. However, almost all MM patients will relapse [9]. This high relapse rate in MM patients has suggested the possibility of a CSC that can drive disease progression.

## Evidence for the multiple myeloma stem cell

### Background

The supposition of a multiple myeloma stem cell (MMSC) has been made for a few decades but identification of the exact cell or population has been difficult to accomplish. Biologically, B cells are derived from the common lymphoid progenitor cell and driven through pro-B to pre-B cell subsets by activation of transcription factors and subsequent expression of the μ chain immunoglobulin and rearrangement of the heavy chain. Development then moves to secondary lymphoid organs (i.e. spleen, lymph nodes) where exposure to antigens induces generation of germinal centers, somatic hypermutation at the Ig locus and proliferation to create clonal-specific memory and short-term and long-term antibody-secreting plasma cells (PCs) that can respond to subsequent antigen exposures. Memory and long-term PCs reside in the BM where they receive support from the BM stroma for survival and activation. The specific cell population, within the B cell/PC lineage, that contains the supposed MM CSC, however, is still unknown.

### Cellular identification

The key cellular component of MM, the monoclonal (M) protein-secreting plasma cell, is a terminally differentiated cell type that arises from the B cell lineage. The pathological highlights of MM suggest, however, that malignancy is incurred in B cells and not in the plasma cell population. Early studies demonstrated that in MGUS and MM patients, a fraction of B cells, branded as clonotypic B cells, were present at differentiated states; though this population exhibited heterogeneity [10,11]. Additionally, Bergsagel *et al*. identified these clonotypic cells in assorted numbers among patients, with steady levels observed during treatment but significantly higher levels during relapse states [12]. These circulating B cells also had chromosomal aberrations and Ig rearrangements particular to a certain idiotype seen in the malignant PC [13-15]. These cells also could differentiate into antibody secreting plasma cells suggesting that the progenitor population of myeloma is contained in the B cell fraction and has progenitor-like characteristics [16]. Further studies of these B cells identified somatic hypermutation in the VDJ region of the genome with deficiency of intraclonal variation suggesting a post-germinal center B cell [10,17]. Phenotypic studies of these circulating clonotypic B cells demonstrated that they resembled memory B cells, a post-germinal center, pre-plasma cell generated to establish long-term immunity [18]. This property along with the ability to self-renew suggested that this phenotype may be the population containing the malignant myeloma stem cell but the research by Rasmussen *et al*. [18] has been the only reported suggestion of memory B cells as the myeloma CSC population.

### Biological characteristics

Biological activity of the proposed MMSCs has been variable due to the plasticity of the surface markers and the assay used to determine clonogenic activity. This has led to some confusion regarding the surface marker phenotype of the MMSC population (summarized in Table 1). The first model to understand the biology of myeloma stem cells was done by directly injecting myeloma cells from the BM of patients into a subcutaneously implanted human fetal bone chip in SCID mice (named as SCID-hu) [19]. These mice developed clinical characteristics of MM, such as hypercalcemia and circulating M protein. In a later study, it was reported that cells from reconstituted SCID-hu mice were able to engraft secondary SCID recipients, which validates the transferable phenotype of a stem cell population [20]. However, the engrafted population was primarily a CD38++CD45- surface phenotype and no CD19+ B cell was capable of growth [20]. A secondary study has also identified the CD19-CD45-CD38 + CD138+ population as being the tumorigenic stem-like population for MM in another mouse model [21].

**Table 1:** List of cell surface markers utilized to identify proposed multiple myeloma stem cell

**Phenotype**	**References**
CD19 + CD38-CD27+	Rasmussen, T *et al*., Leukemia and Lymphoma 2004 [18]
CD19 + CD138-	Pilarski, LM *et al*., Blood 2000 [22]
	Pilarski, LM *et al*., Exp Hematology 2002 [23]
	Matsui, W *et al*., Blood 2004 [24]
CD19 + CD138-CD27+	Matsui, W *et al*., Cancer Research 2008 [25]
CD138-ALDH+	Reghunathan, R *et al*., Oncotarget 2013 [26]
	Matsui, W *et al*., Cancer Research 2008 [25]
CD19 + CD34-Lchain(λ) + ALDH+	Boucher, K *et al*., Clinical Cancer Research 2012 [55]
CD38 + CD45-	Yaccoby, S *et al*., Blood 1999 [20]
CD19-CD45-CD38 + CD138+	Kim, D *et al*., Leukemia 2012 [21]

In stark contrast though, Pilarski *et al*. isolated clonotypic circulating B cells from a progressed MM patient, with the ability to engraft immunodeficient mice and demonstrating clinical phenotypes of lytic bone disease and the presence of circulating M-protein [22]. Furthermore, this leukemic B cell had Ig rearrangements identical to the CD138+ plasma cell, suggesting the involvement of the CD19+ B cell as the progenitor population [14]. The ability to transfer into secondary recipients was not performed, however. Nonetheless, this study was subsequently followed to better ascertain the surface marker phenotype of the myeloma CSC. CD19+ cells lacking the plasma cell marker, CD138/syndecan1, were able to give rise to the tumor population in NOD/SCID mice generating both CD19+ and CD138+ myeloma cells [23]. This further suggested that the myelomagenic population was contained in the B cell lineage but not in the plasma cell pool.

Another study confirmed the lack of CD138 expression in the MMSC phenotype, as primary myeloma BM samples expressing CD138+ were unable to engraft NOD/SCID mice, with the engraftment potential contained solely in the CD138- cell population, incurring plasma cell proliferation and inducing production of M protein *in vivo*[24]. *In vitro* colony forming assays further demonstrated the clonogenic capacity of CD138- and not CD138+ cells validating the *in vivo* transplantation studies. Additional studies to identify the cell surface subset of myeloma CSCs found that the cells resembled a memory B subset in that the clonogenic population expressed CD19 + CD27 + CD138 [25]. This population, obtained from peripheral blood of myeloma patients, engrafted NOD/SCID animals and was transferrable to secondary recipients as the CD19+ cells from the BM of the primary mice engrafted. Additionally, the potential validity of CD138- MMSC and “stemness” has been described by Reghunathan *et al*. using human MM cell lines to demonstrate the CD138- MMSC neoplasticity [26].

Though research suggests conflicting subsets in identification of the MMSC, the issue may be simply due to the source of the cells, the isolation procedure of these cells or the *in vivo* or *in vitro* assay used to determine potential clonogenicity and progenitor status. Collectively, however, the MMSC population appears to reside in the B cell lineage but not in the plasma cell pool.

### Signaling pathways

CSCs utilize many of the pathways that regulate and maintain normal stem cells, adapting the ability to self-renew to maintain the malignancy. A feature identified from embryonic stem cells (ESC) to HSCs is the use of pathways established in many developmental mechanisms, including Hedgehog (Hh), Wnt and Notch pathways. Early reports demonstrated the role of these pathways in a number of cancers establishing the manipulation of self-renewal mechanisms by malignant cells to continue disease progression [27-30].

Hedgehog signaling was the first to be implicated in the maintenance of MM CSCs demonstrating overexpression in the pathway both from human myeloma cell lines and primary human myeloma samples [31]. Biologically, Hedgehog is involved in stem cell maintenance of ESCs and utilizes a ligand-receptor mechanism of Hh to Patched 1 (Ptch1) to the receptor Smoothened to induce activation of the pathway [32]. Cyclopamine, which targets and inhibits Smoothened, was found to induce apoptosis in MM cells [31]. The use of cyclopamine in specifically treating cancer stem cells was demonstrated in lung and prostate cancer studies, inducing apoptosis and inhibiting growth of the malignant cells [33,34]. One clinical trial utilizing a small molecule inhibitor of the Hedgehog pathway demonstrated significant responses in over 50% of advanced metastatic basal cell carcinoma patients [35]. Another clinical use of this molecule was performed in a medulloblastoma patient with some success [36]. However, the clinical use of Hedgehog inhibitors in treating MM has not been reported.

The Wnt pathway utilizes 19 conserved glycoproteins that bind to the transmembrane receptor Frizzled, activating canonical β-catenin signaling and noncanonical pathways to induce proliferation and activation [37]. In fact, genetic manipulation of normal Wnt signaling affects the development and function of multiple organs [38]. Aberrant activation of the Wnt pathway promotes proliferation of both MM cell lines and primary patient samples [39]. This activation is induced by intracellular mechanisms and through crosstalk with the BM microenvironment [40-43]. Small molecule inhibitors of the Wnt pathway have disrupted the maintenance of MM cells both *in vitro* and *in vivo* providing the possibility of developing Wnt-targeted inhibitors for clinical treatment of MM [44,45] gp96 is a molecular chaperone in the endoplasmic reticulum and it is regulated by the unfolded protein response pathway [46]. It was shown recently that gp96 is a critical chaperone for Wnt co-receptor LRP6 [47]. Targeted inhibition of gp96 genetically and pharmacologically has shown to be an effective strategy against multiple myeloma through inhibition of Wnt-LRP6-surivivin pathway [48].

The Notch signaling pathway is involved in various cellular events from proliferation, differentiation and apoptosis to cell maintenance and survival [49]. The expression of Notch in stem-like populations in various cancers promotes survival of the CSC and progression of disease [50-54]. Activation of Notch in MM promotes proliferation and induces enhanced development of the disease [55-57]. One study investigated the expression of Notch on BM clonotypic B cells from MM patients and found high expression of Notch on these cells indicating an involvement of Notch signaling in maintaining the MMSC [58]. Inhibitors of Notch signaling have successfully prevented localization and migration of MM cells to the BM and induced apoptosis of these cells but this has not been attempted in clinical settings [59-61].

These studies underlie the fact that CSCs, and more specifically, MMSCs, utilize mechanisms similar to normal stem cells to survive, maintain disease and increase progression of disease. Identifying the specific MMSC population that maintains MM would be advantageous in developing a therapy unique to targeting these cells.

## Recurrence and disease progression

### Current therapeutics

Treatment of MM has largely been established through utilization of chemotherapy. Weder in 1950 published the first successful treatment of MM through the use of urethane, an ethyl carbamate that had little success in treating other hematological malignancies such as leukemia [62]. The use of melphalan and cyclophosphamide and the corticosteroids prednisone and dexamethasone enhanced the treatment of MM and the subsequent combination of these drugs has improved the treatment response rate and the disease progression-free survival [63-66]. However, these treatments still did not produce greater overall survival and long-term remission, requiring the development of better therapies and treatments [67]. Immunomodulatory agents, thalidomide and lenalidomide, both have had promising results in improving time to remission and survival in newly diagnosed and relapsed MM [68,69]. Proteasome inhibitors such as bortezomib and carfilzomib are effective in the treatment of multiple myeloma. Autologous hematopoietic stem cell transplantation has also been used as therapy in healthy, fit patient population. Induction chemotherapy combining 2–3 drugs with immunomudulatory agent, proteasome inhibitor and corticosteroid, followed by autologous SCT and post-transplant maintenance therapy has become a gold standard of therapy for myeloma. This approach has significantly improved the response rate and patients’ overall outcome. Other novel agents have also emerged for the treatment of multiple myeloma including HSP90 inhibitors [70], Bruton’s tyrosine kinase (BTK) inhibitors [71], and novel immunomodulating agents [72,73]. Unfortunately these therapies have been unable to provide complete eradication of disease. Nearly all MM patients will eventually relapse and become resistant to currently available chemoagents. The exact cause for the relapse remains to be defined and may vary between individual patients. One of the possibilities for the relapse is the persistence of a CSC in myeloma [9]. Many mechanisms involving CSC dormancy have been proposed including evasion of anti-tumor immunity by the tumor cell, the ability of the immune system to control residual tumor cells and the lack of an optimal microenvironment for growth [74].

### Microenvironment and drug resistance

**Figure 1:**
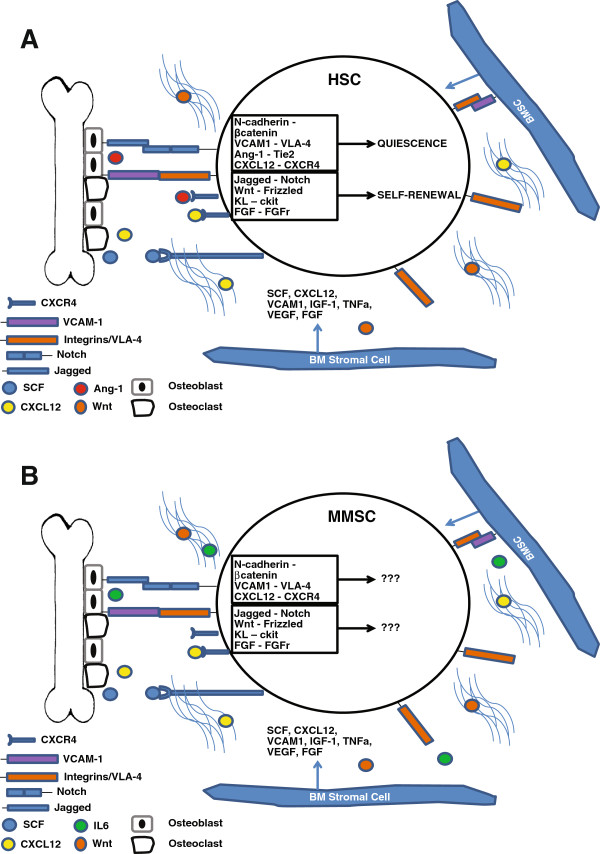
**Similarities of hematopoietic stem cells and multiple myeloma stem cells.** The bone marrow microenvironment of normal **(A)** and multiple myeloma **(B)** and the signaling involved in maintaining the cell populations at the niche.

The capacity for the CSC to remain dormant at length requires interaction with a specialized niche or microenvironment for optimal support. It is unknown whether MMSC resides in BM or in the post-germinal center of secondary lymphoid organs. It was postulated that similar to B cells and plasma cells, MMSCs are maintained at specific niches in the BM for a long-term survival [75,76]. The BM microenvironment is characterized by extracellular matrix (ECM) components, including collagens, fibronectin and laminin and cellular parts including hematopoietic cells, BM stromal cells, BM endothelial cells, osteoblasts and osteoclasts [76-78] (See Figure 1). Extracellular signaling and cell-cell interactions maintain the homeostatic environment and contribute to MM pathogenesis and maintenance. The various BM stromal cells secrete factors including interleukin 6 (IL6), RANK ligand (RANKL), insulin-like growth factor 1 (IGF1), tumor necrosis factor alpha (TNFα), vascular endothelial growth factor (VEGF), B cell activating factor (BAFF) and stromal cell-derived factor 1 alpha (SDF1) which are required for normal cell function and exacerbate MM disease progression [79-83]. A paracrine loop is stimulated in that MM adhesion to the BM niche induces a response by stromal cells to secrete TNFα and VEGF which upregulate IL6 secretion [84-86].

The bone cellular components, osteoblasts and osteoclasts, are involved in MM. Osteoblasts secrete IL6 which induces bone lysis along with MM cell proliferation [87,88]. They also secrete osteoprotegerin (OPG), which prevents TRAIL-mediated death in MM cells [89]. Osteoclasts are involved in bone destruction and seen to be over proliferative in patients [90]. The cells are activated by RANKL, IL-3 and IL-6, all of which are secreted heavily by BM stromal cells [91]. RANK is expressed on osteoclast progenitor cells, inducing their differentiation to osteoclasts and further exacerbating bone lytic damage [92].

Another important mechanism in cell-niche interaction of normal and cancer cells are the family of integrins [93]. Very late antigen 4 (VLA-4), which is composed of α4 and β1, and VLA-5, which includes α5 and β1, are both heavily involved in hematopoietic cell and plasma cell adhesion [94,95]. Interestingly, there is a downregulation of VLA-5 on progressing malignant PCs and an upregulation of VLA-4 [94]. Binding of VLA-4 to the niche induces NFκB activation in MM cells and induces a cell adhesion-mediated drug resistance (CAM-DR) [95]. Interestingly, the overexpression of VLA-4 is found dramatically in resistant plasma cells. CAM-DR, initially identified in MM, has been attributed to other stromal-microenvironment diseases including glioblastoma and acute myelogenous leukemia [96,97]. The SDF-1–CXCR4 signaling pathway also plays a critical role in MM cell maintenance. CXCR4 knockdown prevented MM cell line migration to SDF-1 demonstrating the need for this receptor for proper localization [98,99].

The microenvironment of the BM critically maintains various different cells, such as HSCs and memory B cells, for long-term functional responses. Interestingly, many of the signaling molecules involved in maintaining self-renewal of HSCs are involved in propagation of MM indicating that MMSCs may hijack these pathways to maintain long-term survival and maintenance (Figure 1).

### Immune system and tumor dormancy

Though the mechanisms contributing to CSC dormancy may be collective, the immune system plays an important role in either inducing suppressive function or being inhibited by tumor cells. Tumor dormancy has been recognized in AML, CML and breast cancer, where circulating or residual tumor cells are detected though complete remission by clinical standards has been demonstrated [100-102]. A classic model of studying dormant CSCs has been with the DA1-3B Bcr-Abl murine model [103]. Although leukemic cell vaccination by gene transfer (CD80, CD154 and GM-CSF) can induce a tumor dormancy phenomenon compatible with long-term survival, residue disease does persist in a large number of mice [104]. Over time, the lack of sensitivity provided an evasion mechanism inducing a more progressive disease. Further studies have demonstrated that the resistant tumor cells had altered the immune surveillance mechanisms, changing the response by the host immune system and providing protection to targeted cell death [105]. In these tumor studies, blocking CTLA4 enhanced CTL-mediated death of tumor cells, a reason for the development of anti-CTLA4 treatment in the clinic [106]. Specifically, this phenomenon of immune dormancy and CSCs has been demonstrated in colon carcinoma and melanoma [107]. With regard to patients with MM, evidence of immune suppression is abundantly clear from frequent infections to the development of secondary malignancies. MM CSCs may evade the immune system via altering the homeostasis of the various cells in the immune compartments.

### T cells

**Figure 2:**
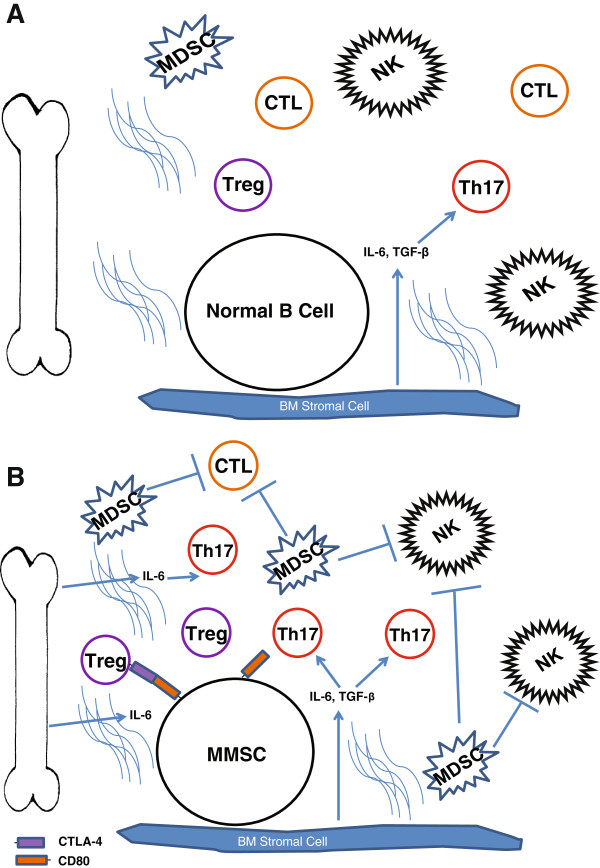
**Bone marrow immunosurveillance and tumor dormancy.** The cellular snapshot of normal **(A)** and multiple myeloma **(B)** bone marrow. The influx of cytokines, primarily IL6, into the stroma induces proliferation of cells involved in suppressing anti-tumor activity.

Early reports have shown that decreases in CD4 and CD8 T cells negatively correlate with survival in MM and loss of T cell function was demonstrated in MM patients indicating an important role for the immune system in MM [108,109]. Of recent note, there have been studies implicating the role of T regulatory (Treg) cells and Th17 cells in MM pathogenesis (Figure 2). The role of Tregs in MM has been conflicting as evidence has pointed to both increased and decreased numbers of Tregs in MM and MGUS patients [109,110]. Though differences in identification of Tregs may contribute to the contradictory results, another study correlated an increased number of Tregs with advanced pathology and disease progression providing a link between Treg numbers and disease status [111]. A study into Th17 cells in the BM of MM patients found there was a significant increase in numbers compared to the peripheral blood (PB), a result not seen in MGUS patients [112]. Elevated levels of Th17 cells can induce myeloma cell proliferation *in vitro* and *in vivo* and the increased levels of IL6 and TGFβ, typically observed in MM patients, can contribute to the differentiation and proliferation of Th17 cells [113]. These data suggest that T cells may contribute to the high frequency of relapse in MM patients, inhibiting anti-tumor immunity and inducing inflammation and proliferation of cells.

### MDSCs and NKs

Myeloid-derived suppressor cells (MDSC) are an immature myeloid population naturally suppressing natural killer (NK) cells, natural killer T cells (NKT) and anti-tumor activity induced by T cells. Suppression is induced by secretion of ROS, COX-2, nitric oxide, IL6 and IL10, among others. A recent report showed that MDSC populations in BM and peripheral blood of MM patients was dramatically increased compared to control donors, correlating with disease progression [114]. Conversely, NK cells, which have cytotoxic activity on tumor cells, have demonstrated different levels of activity correlating with MM disease stage [115]. In early stages of disease, MM has higher expression of NKG2D ligand such as MHC class I related chain protein A (MICA), inducing NKG2D-triggered cell lysis. However, resistance to NK lytic mechanisms is incurred upon more progressive disease with less surface MICA. To further validate the role of NK cells, another report demonstrated that levels of NKT cells were significantly decreased in advanced stage MM versus early MM, MGUS or control patients [116]. This suggests that the cyto-lytic compartment of the immune system is attenuated as disease becomes more progressive providing further tumor dormancy.

## Conclusion

Many studies have looked into the hypothesis of the MMSC with regard to disease relapse of MM, however with little success. The most significant problem is the difficulty to definitely isolate the MMSC to study the unique biology of this cell type. Until this occurs, researchers are left trying to understand the progression of myeloma without knowledge of the start. Though the complexities of specific translocations and mutations in the genetic structure of B cells contribute to the tumorigenic nature of the MMSC, the cell must reside in a hospitable niche, to sustain long-term survival. Understanding the interaction of the MMSC with the surrounding BM microenvironment will enable us to ascertain the required elements for MMSC maintenance and avoidance from therapies. Furthermore, the evasion from immunosurveillance needs to be better studied to comprehend potential ways of targeting the cell. Interestingly, there are many similarities between the HSC and MMSC concerning extracellular and intracellular receptors and signaling. The understanding we have on the complexities required for self-renewal and maintenance of the HSC in the BM could be applied to the MMSC to potentially identify and eradicate the cancer stem cell from the BM environment. Utilizing human cell products and transferring them into mice does not allow for efficient studying of the host microenvironment, particularly related to the roles of immune system in either controlling or later promoting MM. Studies delving into isolating potential tumorigenic populations in syngeneic but humanized mouse models would provide valuable information to study the role of myeloma disease progression, and put into rest the elusive question of the existence of MMSC.

## Competing interest

The authors declare that they have no competing interest.

## Authors’ contributions

All authors wrote, read and approved the final manuscript.
